# Comparative analysis of the transcription-factor gene regulatory networks of E. coli and S. cerevisiae

**DOI:** 10.1186/1752-0509-2-13

**Published:** 2008-01-31

**Authors:** Lev Guzmán-Vargas, Moisés Santillán

**Affiliations:** 1 Unidad Profesional Interdisciplinaria en Ingeniería y Tecnologías Avanzadas, Instituto Politécnico Nacional, Av. IPN No. 2580, L. Ticomán, México D.F. 07340, México; 2 Centro de Investigación y Estudios Avanzados del IPN, Unidad Monterrey, Av. Cerro de las Mitras No. 2565, Col. Obispado, 064060, Monterrey, Nuevo León, México; 3 Centre for Nonlinear Dynamics in Physiology and Medicine, McGill University, McIntyre Medical Sciences Building, 655 Promenade Sir William Osler, H3G1Y6 Montreal QC, Canada

## Abstract

**Background:**

The regulatory interactions between transcription factors (TF) and regulated genes (RG) in a species genome can be lumped together in a single directed graph. The TF's and RG's conform the nodes of this graph, while links are drawn whenever a transcription factor regulates a gene's expression. Projections onto TF nodes can be constructed by linking every two nodes regulating a common gene. Similarly, projections onto RG nodes can be made by linking every two regulated genes sharing at least one common regulator. Recent studies of the connectivity pattern in the transcription-factor regulatory network of many organisms have revealed some interesting properties. However, the differences between TF and RG nodes have not been widely explored.

**Results:**

After analysing the RG and TF projections of the transcription-factor gene regulatory networks of *Escherichia coli *and *Saccharomyces cerevisiae*, we found several common characteristic as well as some noticeable differences. To better understand these differences, we compared the properties of the *E. coli *and *S. cerevisiae *RG- and TF-projected networks with those of the corresponding projections built from randomized versions of the original bipartite networks. These last results indicate that the observed differences are mostly due to the very different ratios of TF to RG counts of the *E. coli *and *S. cerevisiae *bipartite networks, rather than to their having different connectivity patterns.

**Conclusion:**

Since *E. coli *is a prokaryotic organism while *S. cerevisiae *is eukaryotic, there are important differences between them concerning processing of mRNA before translation, DNA packing, amount of junk DNA, and gene regulation. From the results in this paper we conclude that the most important effect such differences have had on the development of the corresponding transcription-factor gene regulatory networks is their very different ratios of TF to RG numbers. This ratio is more than three times larger in *S. cerevisiae *than in *E. coli*. Our calculations reveal that, both species' gene regulatory networks have very similar connectivity patterns, despite their very different TF to RG ratios. An this, to our consideration, indicates that the structure of both networks is optimal from an evolutionary viewpoint.

## Background

Knowing the complete genome of a given species is just a piece of the information thought to be useful in understanding one of the most complicated and important puzzles in science: *How does a biological system work? *To fully understand the behaviour of an organism, an organ, or even a single cell, we need to understand the underlying gene regulatory dynamics. Nevertheless, given the complexity of even a single cell, answering this question is impossible for the time being.

Recent computer simulations of partial or whole genetic networks have demonstrated network behaviours – commonly called systems or emergent properties – that were not apparent from examination of only a few isolated interactions alone. Moreover, the individual building blocks – such as genes or proteins – in a living organism may not posses the explicit understanding of what they perform in the context of cellular processes. The notion of cellular process as an emergent property of the collection of individual interactions may in fact be a better description of life.

The recent advance in high-throughput techniques in genomics, such as microarrays and DNA automatic sequencing, as well as the development of powerful bioinformatics tools, have rendered an impressive amount of novel biological data. For instance, we now know the genome-wide transcription-factor regulatory networks of various species. Unfortunately, the biological information and the mathematical and computational tools available do not allow the development of detailed dynamical models at this level. An alternative to the dilemma stated in the previous paragraph is to employ the techniques of network theory. Among others, the advantages of network theory are that: it allows the description of a network structure with graph concepts, and reveals organizational features shared with numerous other biological and non-biological networks; it is possible with network theory to quantitatively describe networks of hundreds or thousands of interacting components; and finally, in some cases, the observed network topology gives clues about its evolution, and the observed network organization may help to elucidate its function and dynamic responses [1-7].

In this work we present a comparative analysis of two different genome-wide transcription-factor gene regulatory networks: those of the bacterium *Escherichia coli *and the budding yeast *Saccharomyces cerevisiae*. We measured various network properties for the bipartite networks (with unidirectional links from the transcription factors to the regulated genes), as well as for the networks resulting from projections onto the transcription-factor and onto the regulated-gene nodes. The performed measurements include the clustering coefficient, the degree distribution, the efficiency of information transfer and the network cost. We also constructed randomized networks with the same degree distributions as those of *E. coli *and *S. cerevisiae*, and carried out the same measurements to compare with the original networks. Finally, we tested network robustness by subjecting the original and the randomized networks to removal of the most connected nodes, and seeing to what extent the clustering coefficient changes.

The basic molecular mechanisms involved in gene expression are essentially the same in both prokaryotic and eukaryotic cells. However, there are important differences between them concerning processing of mRNA before translation, DNA packing, amount of junk DNA, and gene regulation. Since *E. coli *is a prokaryotic organism while *S. cerevisiae *is eukaryotic, we investigate in this work the possibility that the above referred differences emerge at the whole network level and can be identified via network theory analysis.

## Results and Discussion

### Global and projected network topology

**Figure 1:**
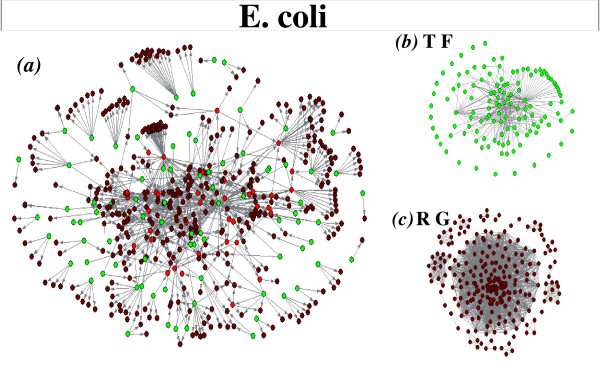
**Representation of the *E. coli *transcriptional regulatory network**. a) Representation of the transcription-factor gene regulatory network of *E. coli*. Green circles represent transcription factors, brown circles denote regulated genes, and those with both functions are coloured in red. Projections of the network onto b) transcription factor and onto c) regulated gene nodes are also shown.

**Figure 2:**
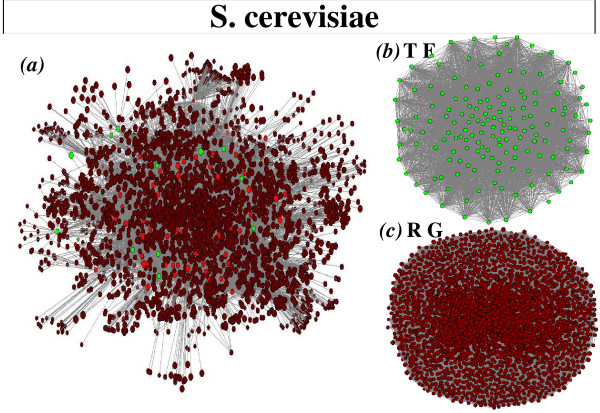
**Representation of the *S. cerevisiae *transcriptional regulatory network**. a) Representation of the transcription-factor gene regulatory network of *S. cerevisiae*. Green circles represent transcription factors, brown circles denote regulated genes, and those with both functions are coloured in red. We also show the network projections onto b) transcription factors and onto c) regulated genes.

The *E. coli *and *S. cerevisiae *gene regulatory networks are bipartite; i.e. they comprise two kinds of nodes, transcription factors (TF) and regulated genes (RG), with the links being directed from the TF to the RG nodes. Bipartite gene regulatory networks can be projected onto either networks comprising only transcription factors or networks comprising only regulated genes. The projections onto transcription factors are constructed by linking every two nodes regulating a common gene; similarly, the projections onto regulated genes are built by linking every two regulated genes sharing a common regulator. The *E. coli *and *S. cerevisiae *original (non-randomized) bipartite and projected networks are respectively pictured in Figures 1 and 2.

**Figure 3:**
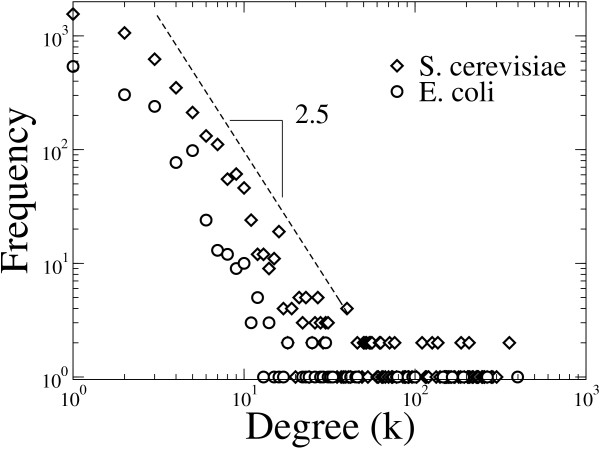
**Degree distribution of bipartite networks**. Connectivity distributions for the *E. coli *and *S. cerevisiae *networks. The dotted line represents an exponent -2.5.

A node's connectivity or degree is defined as the sum of links with one end at the node. In Figure 3, the connectivity distributions are shown for the *E. coli *and *S. cerevisiae *undirected networks. Notice that, in agreement with previous studies [8-10], both distributions show a scale-free behaviour. Previous studies have reported that the outgoing-link distributions show a scale-free behaviour, while the distributions of incoming links show an exponential decay for both organisms, when the regulatory networks are considered as directed [9,10].

Random networks have been widely studied and they usually serve as a reference against which other networks are compared to gather information regarding the node connectivity patterns. With this purpose, we built randomized versions of the *E. coli *and *S. cerevisiae *bipartite networks by randomly reconnecting the network links – following the procedure detailed in Materials and Methods. From the way they are built, the randomized networks have the same number of TF and RG nodes, and each node has the same number of links as in the corresponding original networks.

**Figure 4:**
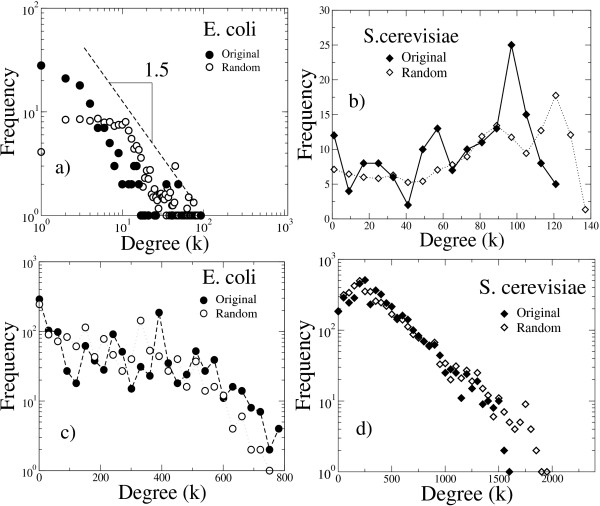
**Connectivity (degree) distributions for the TF- and RG-projected, original and randomized networks of *E. coli *and *S. cerevisiae***. a) *E. coli *TF-projected networks. b) *S. cerevisiae *TF-projected networks. c) *E. coli *RG-projected network. d) *S. cerevisiae *RG-projected networks.

We further calculated the connectivity distributions for the *E. coli *and *S. cerevisiae*, original and randomized, TF and RG projected networks. As seen in the plots of Figures 4a and 4b, the connectivity distributions corresponding to the *E. coli *TF-projected original and randomized networks are power-law distributions with slope about -1.5. The corresponding *S. cerevisiae *distributions show a slight non-monotonic growing tendency.

In the plots of figures 4c and 4d, the connectivity distributions for the *E. coli *and *S. cerevisiae *RG-projected networks are presented. Notice that the connectivity distributions for the *S. cerevisiae *RG-projected networks show an approximately exponential decreasing behaviour, while the distributions corresponding to *E. coli *have various local maxima and present a slow decreasing tendency.

Interestingly, the TF and RG projected networks of *E. coli *and *S. cerevisiae *have very different connectivity structures, despite the strong similarities observed in the bipartite-network link distributions (see Figure 3). Furthermore, the connectivity distributions of the original and randomized RG-projected networks are very similar in both the *E. coli *and *S. cerevisie *cases, while small deviations from the behaviour of the randomized plots are observed in the TF projections. This indicates to our understanding that the observed differences between the connectivity distributions of the *E. coli *and *S. cerevisiae *projected networks are mainly due to the very different number transcription factors and regulated genes in both organisms.

A network's clustering coefficient (*C*) is an estimation of its nodes tendency to form tightly connected clusters (see Materials and Methods). We calculated the clustering coefficient of the *E. coli *and *S. cerevisiae*, original and randomized, TF- and RG-projected networks, and the results are shown in Table 1. Observe that the clustering coefficient of the original and randomized RG projected networks are quite similar for both *E. coli *and *S. cerevisiae*. Contrarily, the *C *values of the randomized TF projections are consistently larger than those of the original network projections.

**Table 1:** Statistics of the transcriptional regulatory networks. In this table we show different statistical properties (like the global communication efficiency, the clustering coefficient, and the cost), measured for the bipartite, TF-projected and RG-projected, original and randomized networks of *E. coli *and *S. cerevisiae*. Subindex *rand *denotes the values corresponding to the randomized networks.

Network	Nodes	Edges	*E*	*E*_*rand*_	C	*C*_*rand*_	*σ*	*σ*_*rand*_
Bipartite network (*E. coli*)	1402	2793	--	--	--	--	--	--
TF projection (*E. coli*)	153	481	0.342	0.515 ± 0.011	0.484	0.642 ± 0.015	0.043	0.104 ± 0.002
RG projection (*E. coli*)	1319	162337	0.501	0.524 ± 0.001	0.863	0.811 ± 0.002	0.181	0.16 ± 0.002

Bipartite network (*S. cerevisiae*)	4441	12853	--	--	--	--	--	--
TF projection (*S. cerevisiae*)	157	5622	0.721	0.755 ± 0.003	0.742	0.807 ± 0.008	0.451	0.518 ± 0.003
RG projection (*S. cerevisiae*)	4410	908227	0.544	0.563 ± 0.005	0.701	0.693 ± 0.004	0.091	0.092 ± 0.001

Following the procedure detailed in Materials and Methods, we calculated the global communication efficiency (*E*) for the *E. coli *and *S. cerevisiae *TF- and RG-projected networks, as well as for their randomized versions. The results are also reported in Table 1. An efficiency close to one means that very short paths can be found communicating any two nodes in the network. Since the projected networks are not fully connected, we calculated *E *for the largest component in each case. In all cases, these largest components comprise the vast majority of the nodes. Notice that the communication efficiencies of the original and randomized RG-projected networks are very similar for both *E. coli *and *S. cerevisiae*. On the other hand, the value of *E *for the TF-projected networks is smaller in the original than in the randomized networks. This is true for both organisms, although the difference is smaller in the case of *S. cerevisiae*. The cost, *σ *associated to a network is defined as the ratio of the current number of links to the maximum possible link count, given the network nodes. We can see in Table 1 that the original and randomized RG projections present very similar network costs for the two studied organisms. In contrast, the original TF-projected networks of both species have smaller cost values than the corresponding randomized projections. Notice that, in both species, the TF-projected networks have fewer links than the corresponding original networks. This is due to the fact that some RGs are only regulated by a single TF and therefore such links are lost when the projection is made.

In summary, we have observed that the RG original and randomized projected networks have very similar properties for both studied organisms. Contrarily, consistent differences were observed between the corresponding original and randomized TF projections: *E < E*_*rand*_, *C < C*_*rand*_, and *σ *<*σ*_*rand*_.

Furthermore, although observed in both organisms, these differences are smaller in the *S. cerevisiae *case. To our consideration, this symmetric behaviour, together with the fact that the distributions of links incoming to the RG and outgoing from the TF nodes are alike in both species, indicates that the transcription-factor regulatory networks of *E. coli *and *S. cerevisiae *obey similar connection patterns, and that the most important dissimilarity between them is their very different number of regulated genes and transcription factors.

### Network projections and levels of co-regulation interaction

We have seen that the clustering coefficient, the efficiency, and the cost of the original TF-projected networks are consistently smaller than those of the corresponding randomized networks. However, the TF projections can be constructed using different rules, and this may affect the above behaviour. For instance, a more restrictive rule consists of drawing a link between two TFs only if they share *g*_*S *_target genes or more, with *g*_*S *_> 1. If, when using this new rule, a high clustering coefficient is observed for high values of *g*_*S*_, it indicates a strong tendency to co-regulation.

Starting with the original networks and their corresponding randomized versions, we constructed TF projections for different values of *g*_*S*_, and compared their topological properties. The first thing we observed is that, as *g*_*S *_increases, the number of links in the projected networks decreases. That is, the number of TF pairs sharing at least *g*_*S *_target genes is a decreasing function of *g*_*S*_.

**Figure 5:**
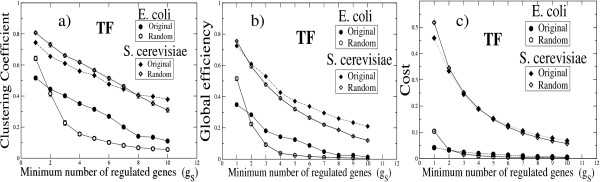
**Topological properties and level of co-regulation interaction**. Plots of the **(a) **clustering coefficient (*C*), **(b) **efficiency (*E*) and **(c) **cost (*σ*) against the minimum number of common regulated genes (*g*_*S*_) required to draw a link between to nodes in the TF-projected network. We observe that for *g*_*S *_equal to one and for the two species, a higher value is assigned to *C*, *E *and *σ *of randomized networks in comparison with the original TF's networks. However, while for *E. coli *network *C*, *E *and *σ *show a decreasing behavior as *g*_*S *_increases, these quantities in the randomized version show a faster decay, such that for *g*_*S *_> 2, they become smaller than the corresponding values of the original networks. For random projected networks, we show mean values ± standard error from 10^2 ^independent realizations.

In Figure 5 we show how the clustering coefficient, the efficiency, and the cost of the TF projections depend on *g*_*S*_. Observe that, for *g*_*S *_= 1, all these quantities achieve higher values in the randomized than in the original networks, as discussed above. Furthermore, in the *E. coli *network, the clustering coefficient,

the efficiency, and the cost decrease with *g*_*S*_, and this decay is faster for the randomized networks; thus, *C > C*_*rand*_, *E > E*_*rand*_, and *σ *> *σ*_*rand *_for *g*_*S *_> 2. This finding reveals that the transcriptional regulatory organization has not arisen by chance and is determined by different levels of co-regulation. In contrast, in *S. cerevisiae*, all the three monitored quantities also decrease monotonically as *g*_*S *_increases, but the values corresponding to the original and randomized networks are consistently close each other.

### Network robustness to directed attacks and random failures

Recent studies suggest that a network's connectivity pattern determines its robustness to external perturbations, such as removal of nodes or links. To test this, we measured the effects of directed attacks and random failures on the network organization. These measurements were carried out as follows:

1. A given fraction of either TF or RG nodes was eliminated from the original and the randomized *E. coli *and *S. cerevisiae *bipartite networks. The nodes to be removed were either chosen as the most connected ones (directed attacks), or at random (random failures).

2. The networks' emerging organization was evaluated by calculating their clustering coefficient.

3. The whole process was repeated for several fractions of removed nodes.

**Figure 6:**
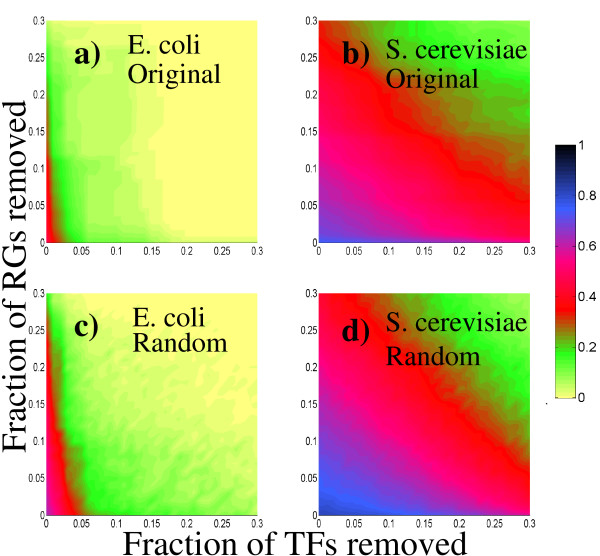
**Clustering-coefficient profiles for the TF-projected networks under directed attacks to the best connected transcription factors and regulated genes**. In these panels, we show the average clustering coefficient value of TF-projected networks after attacks according to the color scheme shown in the bar. a) *E. coli *original network. b) *S. cerevisiae *original network. c) *E. coli *randomized network. d) *S. cerevisiae *randomized network. We remark the slightly slower decay of the clustering coefficient in the randomized case of *E. coli *TF network. For S. cerevisiae network no appreciable differences are observed between the original and randomized cases.

In Figure 6 we illustrate the effect of directed attacks on the clustering coefficient of the TF projected networks. Observe that, for *E. coli*, both the original and the randomized networks exhibit a similar profile, except for a slightly slower decay of the clustering coefficient in the randomized network. On the other hand, no appreciable difference can be observed between the plots corresponding to the original and randomized networks of *S. cerevisiae*. Finally, when the *E. coli *and *S. cerevisiae *networks are compared, we see that the *E. coli *networks are more robust to attacks on the regulated genes than they are to attacks on the transcription factors, while the *S. cerevisiae *networks are a little less robust than those of *E. coli *RG attacks, but they are much more robust to TF attacks.

**Figure 7:**
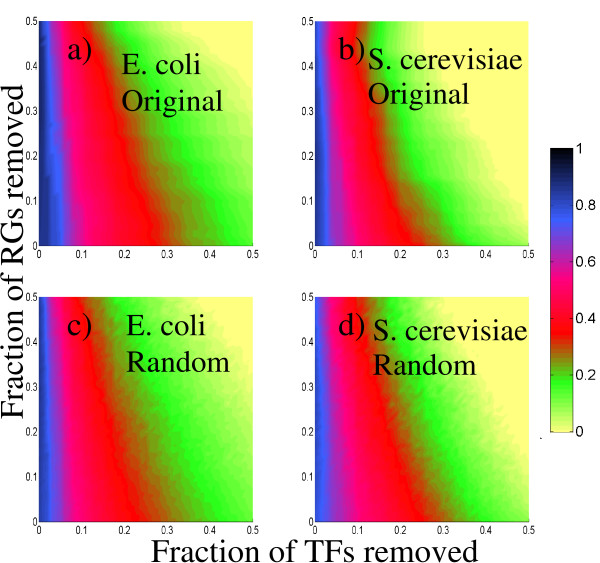
**Clustering-coefficient profiles for the RG-projected networks under directed attacks to the best connected transcription factors and regulated genes**. We show the clustering coefficient value according to the color bar. a) *E. coli *original network. b) *S. cerevisiae *original network. c) *E. coli *randomized network. d) *S. cerevisiae *randomized network. We observe that no appreciable differences are present between original and random networks. Remarkably, both organisms organisms are more robust against RG attacks than they are to TF attacks.

The robustness of the RG-projected networks' clustering coefficient to directed attacks is pictured in Figure 7. Contrarily to the TF projections, there is no appreciable difference between the robustness of the original and the randomized networks, as well as between the *E. coli *and *S. cerevisiae *networks. In all cases, the networks are noticeably more robust to RG attacks than they are to TF attacks.

Our calculations reveal that random removal of nodes (random failures) has almost no effect on the *E. coli *and *S. cerevisiae *RG- and TF-projected networks, both original and randomized: the clustering coefficient remains quite similar to its initial value even when 30% of TF or RG nodes are removed from the corresponding networks (data not shown). This behaviour is in agreement with the fault tolerance properties that characterize scale-free networks [11].

## Conclusion

We have carried a comparative analysis of the transcription-factor gene regulatory networks of *E. coli *and *S. cerevisiae*. This analysis consisted in measuring a number of statistical properties on the TF and RG projections of both networks, as well as on randomized versions of them. Some interesting observations arising from these measurements are:

• The ratio of transcription factor to regulated gene number is about 0.116 in *E. coli*, and about 0.036 in *S. cerevisiae*.

• The distributions of link counts of the *E. coli *and *S. cerevisiae *bipartite networks are very much alike; they can be approximately fitted by a decreasing power-law function.

• The connectivity distributions of the *E. coli *and *S. cerevisiae *RG-projected networks are very different, as are the connectivity distributions of the corresponding TF projections.

Intriguingly, the connectivity distributions associated to the projected networks of *E. coli *are quite different to those corresponding to *S. cerevisiae*; whereas the connectivity distributions of the original bipartite networks are alike. A possible explanation for these differences is that the nodes of the *E. coli *and *S. cerevisiae *networks have different connection patterns. However, when the same measurements were carried out on networks preserving the number of RG and TF nodes, as well number of links incoming and outgoing from each node, but in which the links have been randomly reconnected, we observed that their projections have connectivity distributions very similar to those of the corresponding original networks. Therefore, we conclude that the above mentioned differences are mostly due to the very dissimilar ratios of RG to TF numbers the *E. coli *and *S. cerevisiae *networks have.

We also measured the clustering coefficient (*C*), the communication efficiency (*E*), and the cost (*σ*) of the RG- and TF-projected networks of *E. coli *and *S. cerevisiae*, both original and randomized. The values of all these quantities associated the *E. coli *networks differ from those of *S. cerevisiae*. However, the *E*, *C*, and *σ *values of both original RG projections are very similar to those of their randomized counterparts. Moreover, the following relations are satisfied for the TF projections of both species: *E < E*_*rand*_, *C < C*_*rand*_, and *σ *> *σ*_*rand*_. Recall that the randomized networks have the same number of TF and RG nodes, as well as the same number of links for every node.

When a more restrictive rule was used to perform the projections onto the TF nodes, we observed important differences between the original networks and their randomized counterparts, for both species. These results suggest that the transcriptional regulatory networks involve different levels of co-regulation. Finally, in agreement with the assertion above, the RG- and TF-projected networks of both species show similar robustness properties to directed attacks on the RG and TF nodes.

In all the above discussed results, we have seen that the RG-projected, original and randomized networks have very similar behaviours, for both *E. coli *and *S. cerevisiae*. In contrast, the properties of the original TF-projected networks deviate from those of their randomized counterparts, but these deviations are relatively small, and they are of the same kind in *E. coli *as well as in *S. cerevisiae*. To our consideration, these coincidences reinforce our previous assertions that the differences observed between the *E. coli *and *S. cerevisiae *networks are mainly due to their very dissimilar ratios of RG to TF numbers, and not to their nodes having very different connection patterns. Moreover, the fact that the TF original projections are consistently different from their randomized versions indicates, to our consideration, that the development of the TF connection patterns has been subject to strong evolutionary stresses, contrarily to those of the regulated genes.

*E. coli *is a prokaryotic organism while *S. cerevisiae *is eukaryotic. This means that important differences can be observed between them regarding processing of mRNA before translation, DNA packing, amount of junk DNA, and gene regulation. From the results described above we conclude that the most important effect such differences have had on the development of the corresponding transcription-factor gene regulatory networks is their very different ratio of TF to RG counts: it is more than three times larger in the *S. cerevisiae *than in the *E. coli *networks. Our calculations reveal that, both species' gene regulatory networks have very similar connection patterns between the RG and TF nodes, despite their very different numbers.

## Methods

The interaction dataset for the transcription-factor regulatory network of *E. Coli *was obtained from the RegulonDB database [12]. For *S. cerevisiae *we used the data described in [13]. For *E. Coli*, the resulting network has 1402 genes, with 153 regulatory genes and 1319 regulated genes. In *S. cerevisiae*, the resulting network has 4441, genes with 157 transcription factors and 4410 regulated genes. The transcription-factor gene regulatory networks of *E. coli *and *S. cerevisiae *are bipartite because they consist of two different kinds of nodes: transcription factors (TF) and regulated genes (RG), with the links directed from the TF to the RG nodes. The bipartite networks can be either projected onto networks comprising only transcription factors, or onto networks comprising only regulated genes. The projections onto transcription factors are constructed by linking every two nodes regulating one common gene at least; similarly, the projections onto regulated genes are made by linking every two regulated genes sharing one common regulator at least. Randomized version of the bipartite networks were built using the following algorithm:

• Given a bipartite network, we made a list of all the RG nodes, repeating each node as many times as the number on links incoming to it in the bipartite network. A similar list was made for the TF nodes. From the way they were constructed, the number of elements in these lists equals the total number of links in the bipartite network.

• One RG and one TF were selected and eliminated from the lists above, and a link was established between these RG and TF nodes in the new randomized bipartite network. This step was repeated iteratively until the lists built in the above step are empty.

The randomized networks constructed in this way have the same number of RG and TF nodes, and each node has the same number of links incoming or outgoing from it, as the corresponding original network. A node's clustering coefficient is by definition the probability that every two of its nearest neighbours are connected between them. Thus, the the clustering coefficient can be calculated as the number of triangles with one vertex in the node divided by the total number of nearest neighbours couples. If *g*_*i *_is the number of links connecting *k*_*i *_neighbours of a node *i*, then, the node clustering coefficient is given by:

*C*_*i *_= 2*g*_*i*_/(*k*_*i*_(*k*_*i *_- 1)),

where *k*_*i*_(*k*_*i *_- 1)/2 is the maximum possible number of links between *k*_*i *_nodes. The network average clustering coefficient was calculated by averaging over all the network nodes.

The communication efficiency of a network was introduced to quantify the idea of parallel information transfer in a complex networks [14]. The global efficiency is defined as:

$$E=\frac{1}{N(N-1)}\sum_{i\neq j}\frac{1}{l_{ij}},$$

where *l*_*ij *_is the minimum path length connecting nodes *i *and *j*.

The cost of a complex network with *N *nodes is defined as the ratio of the actual number of links to the maximum possible number of links between the network nodes (*N*(*N *- 1)/2).

## Authors' contributions

LG carried out the calculations, performed the statistical analysis and drafted the manuscript. MS participated in the design of the study, participated in its coordination and drafted the manuscript. All authors read and approved the final manuscript.
